# LRRC56 deficiency cause motile ciliopathies in humans and mice

**DOI:** 10.3389/fgene.2025.1658063

**Published:** 2025-12-17

**Authors:** Xiao-Hui Xie, Heng Gu, Jun-Lin Yang, Zhuang-Zhuang Yuan, Ke-Le Qin, Zhi-Ping Tan

**Affiliations:** 1 Department of Cardiovascular Surgery, The Second Xiangya Hospital, Central South University, Changsha, China; 2 Clinical Center for Gene Diagnosis and Therapy, Department of Cardiovascular Surgery, The Second Xiangya Hospital of Central South University, Changsha, China; 3 Department of Cell Biology, School of Life Sciences, Central South University, Changsha, China; 4 The Second Department of Thoracic Surgery, Affiliated Cancer Hospital of Xiangya School of Medicine, Central South University, Changsha, China; 5 Center for Medical Research, Pingshan District Central Hospital of Shenzhen, Guangdong, China

**Keywords:** LRRC56, mice, motile cilia, mutation, primary ciliary dyskinesia

## Abstract

**Introduction:**

Motile ciliopathies represent a group of disorders caused by impaired motility of cilia and flagella, resulting in clinical manifestations such as laterality defects, asthenospermia, chronic respiratory infections, and hydrocephalus. Although nearly 53 genes have been implicated, the genetic etiology remains unresolved in approximately 30% of cases, and the regulatory mechanisms of motile ciliogenesis are still incompletely understood.

**Methods:**

Whole-exome sequencing was employed in a patient with laterality defects and recurrent pulmonary infections. Nasal epithelial brushings underwent high-speed video microscopy (HSVM) to evaluate ciliary beat pattern, and conventional transmission electron microscopy (TEM) to assess ultrastructure. CRISPR/Cas9 technology was harnessed to generate precise *Lrrc56* knockout mice. Quantitative proteomics was conducted on *Lrrc56*-deficient compared with wild-type tissues to explore the mechanisms.

**Results:**

Whole exome sequencing identified a novel homozygous frameshift variant in *LRRC56* (c.148delG, p. Val50Trpfs*22) in the proband. HSVM of nasal cilia from the proband demonstrated severely dyskinetic motion, despite the absence of obvious ultrastructural defects on conventional TEM. *Lrrc56*-knockout mice recapitulated the patient’s laterality defects and also exhibited additional phenotypes consistent with motile ciliopathies, including male infertility, hydrocephalus, and defective mucociliary clearance. Quantitative proteomic analysis revealed markedly reduced expression of cilia-associated proteins, particularly microtubule inner proteins (MIPs) and axonemal dynein assembly factors, in *Lrrc56*-deficient tissues compared with wild-type controls.

**Discussion:**

Our findings establish *LRRC56* as an essential regulator of ciliary motility and highlight its role in the pathogenesis of motile ciliopathies.

## 1 Introduction

Cilia are evolutionarily conserved organelles that extend from the surface of nearly all mammalian cells and are broadly classified as either motile or non-motile (Satir and Christensen, 2007). Motile cilia generate directional fluid flow across epithelial surfaces and play essential roles in embryonic left-right axis patterning, mucociliary clearance, and cerebrospinal fluid circulation (Fliegauf et al., 2007; Brooks and Wallingford, 2014). Structurally, most motile cilia possess a highly organized “9 + 2” axonemal architecture, consisting of nine peripheral microtubule doublets surrounding a central pair, accompanied by accessory components including nexin links, radial spokes, and inner and outer dynein arms (Loreng and Smith, 2017). Disruption of any of these structures can impair ciliary beating and lead to a group of disorders collectively termed motile ciliopathies, which include but are not limited to primary ciliary dyskinesia (PCD), a condition characterized by recurrent respiratory infections, situs abnormalities, infertility, and hydrocephalus (Horani and Ferkol, 2021).

Over the past decade, advances in genomic technologies have enabled the identification of more than 50 genes associated with motile ciliopathies (Lucas et al., 2020). However, up to 30% of clinically diagnosed cases still lack a definitive molecular diagnosis, underscoring the incomplete understanding of axonemal assembly, dynein arm transport, and protein docking mechanisms in motile cilia (Knowles et al., 2016).

Leucine-rich repeat-containing 56 (LRRC56) encodes a relatively uncharacterized protein containing multiple LRR domains. Notably, *LRRC56* has recently been identified as a causative gene for PCD. Individuals with biallelic *LRRC56* variants present with chronic respiratory infections and laterality defects. High-speed video microscopy in that study revealed abnormal ciliary beating, whereas conventional TEM showed no overt ultrastructural abnormalities, underscoring the critical role of LRRC56 in motile ciliary function (Bonnefoy et al., 2018). In this study, we report a novel homozygous *LRRC56* frameshift variant identified in a patient with laterality defects and chronic respiratory symptoms. To investigate the pathogenic mechanisms associated with LRRC56 deficiency, we established a *Lrrc56*-knockout mouse model that recapitulated multiple cardinal phenotypes characteristic of motile ciliopathies. Additionally, quantitative proteomic analysis revealed altered expression of key ciliary proteins and perturbed signaling pathways in mutant tissues. These findings provide mechanistic insight into *LRRC56*-related motile ciliopathy and broaden the spectrum of known genetic etiologies underlying ciliary motility disorders.

## 2 Materials and methods

### 2.1 Subjects

A female patient presenting with clinical features suggestive of motile ciliopathy and her family members were recruited from the Second Xiangya Hospital of Central South University (Changsha, China). Comprehensive clinical evaluations were performed, including assessment of respiratory symptoms, chest X-ray or computed tomography (CT) imaging to detect laterality defects, measurement of nasal nitric oxide (nNO) levels, peripheral blood collection for genetic analysis, and nasal brush biopsies for cellular, molecular, and ultrastructural examination of ciliated respiratory epithelial cells. The clinical diagnosis of motile ciliopathy was based on established international diagnostic guidelines for ciliary disorders (Lucas et al., 2017). This study was approved by the Institutional Ethics Committee of the Second Xiangya Hospital of Central South University. Written informed consent was obtained from all participants.

### 2.2 Whole exome sequencing and bioinformatics analysis

Peripheral blood was obtained from the proband and her immediate family members for genomic DNA isolation, utilizing the DNeasy Blood and Tissue Kit (QIAGEN) according to established protocols. Whole-exome sequencing, including library construction, exome enrichment, sequencing, and bioinformatics analysis, was performed by BerryGenomics Bioinformatics Institute (Beijing, China). Exonic sequences were selectively enriched from genomic DNA using the Agilent SureSelect Human All Exon V6 system (Cat. G9706K), and subsequently subjected to sequencing on the Illumina HiSeq 4000 platform. After quality control, sequencing data were mapped to the GRCh37/hg19 reference genome using Burrows–Wheeler Aligner (BWA). ANNOVAR was utilized for variant detection and annotation.

Variants were prioritized using a multi-step filtering strategy: (1) variants exhibiting a minor allele frequency (MAF) ≥1% in public databases (1000 Genomes, gnomAD) or internal databases were excluded; (2) non-coding and deep intronic variants were eliminated; (3) synonymous substitutions were filtered out; (4) homozygous variants within regions of autozygosity defined by AutoMap were retained; and (5) variants within a curated gene panel previously associated with motile ciliopathies were selected for further analysis.

### 2.3 Sanger sequencing

Candidate variants identified through whole-exome sequencing were confirmed by Sanger sequencing. Primers for each targeted region (listed in Supplementary Table S1) were designed, and bidirectional sequencing was conducted via the dideoxy termination method, employing an ABI 3730xl DNA analyzer (Applied Biosystems).

### 2.4 High-speed video microscopy (HSVM) analysis

Nasal epithelial cells were obtained via brush biopsy from the proband and immediately prepared for HSVM analysis. Samples were visualized using an Olympus BX53 microscope equipped with a ×40 objective. High-speed video was captured at 500 fps at 37 °C using a Prime BSI™ sCMOS camera (Photometrics). Only clearly visible ciliated borders exceeding 50 µm in length and free from mucus contamination were analyzed. And at least 7–10 clearly delineated ciliated strips per sample were analyzed. Automated software (CiliarMove v1.0, Microsoft) was employed to quantify the ciliary beat frequency (CBF).

### 2.5 Generation of *Lrrc56*-knockout mice

The murine *Lrrc56* gene contains 12 exons spanning approximately 1659 bp. *Lrrc56*-knockout (KO) mice were generated using CRISPR-Cas9 genome editing technology (Cyagen Biosciences). PCR genotyping was performed using specific primers: knockout allele primers (F1: 5′-AAC​CAC​GAA​GGA​GAG​AAT​CTT​GAT​G-3′, R1: 5′-CCT​GGC​TTA​ATT​CCG​TGT​CTC​TAT-3′) and wild-type allele primers (F2: 5′-CAT​GGT​TGC​AAC​TCT​CCC​TTT​TC-3′, R1 as above).

All mice were anesthetized by intraperitoneal injection of sodium pentobarbital (50 mg/kg body weight) prior to surgical interventions or tissue collection. For euthanasia, animals were deeply anesthetized using an overdose of sodium pentobarbital (150 mg/kg body weight) followed by cervical dislocation to ensure humane endpoint compliance. All animal procedures adhered strictly to the guidelines outlined by the Institutional Animal Care and Use Committee (IACUC) of Central South University and were in accordance with the NIH Guide for the Care and Use of Laboratory Animals.

### 2.6 Immunofluorescence staining

Bronchial epithelial tissues from mice were harvested, fixed overnight in 4% paraformaldehyde at 4 °C, and processed for immunofluorescence as previously described (Smith et al., 2012). Tissue sections were incubated with primary antibodies against LRRC56 (HPA038728, Sigma-Aldrich, dilution 1:100) and α-tubulin (T7451, Sigma-Aldrich, dilution 1:100) at 4 °C overnight. Sections were subsequently incubated with secondary antibodies: Alexa Fluor 488-conjugated anti-mouse IgG (Invitrogen, dilution 1:500) and Alexa Fluor 555-conjugated anti-rabbit IgG (Invitrogen, dilution 1:500) at 37 °C for 2 h. Nuclei were stained with DAPI (Invitrogen) for 5 min at room temperature. Images were captured using an Olympus BX53 fluorescence microscope and analyzed using cellSens Dimension software (Olympus).

### 2.7 Immunohistochemistry (IHC)

Paraffin-embedded sections were deparaffinized, rehydrated, and underwent antigen retrieval in citrate buffer (pH 6.0). Sections were then incubated with 5% serum blocking solution for 30 min at room temperature, followed by overnight incubation at 4 °C with primary antibodies. After washing, secondary antibody incubation was performed at room temperature for 1 h, followed by visualization with diaminobenzidine (DAB). Sections were counterstained with hematoxylin, dehydrated, and mounted. Antibody details are provided in Supplementary Table S2.

### 2.8 Sperm motility and sperm count assays

Cauda epididymides were isolated from 8-week-old male mice. Spermatozoa were released into phosphate-buffered saline (PBS; Gibco, C14190500BT) by making incisions and incubating at 37 °C for 10 min. Sperm motility was examined using a microscope equipped with a 20× phase-contrast objective, and sperm movements were recorded with a charge-coupled device (CCD) camera. Computer-assisted semen analysis (CASA) was conducted using the Minitube Sperm Vision Digital Semen Evaluation System (Minitube Group, Tiefenbach, Germany) and CASA software (CASA, China). For sperm counts, suspensions were diluted (1:100), and quantification was performed using a hemocytometer.

### 2.9 Histological analysis

Testes were collected after euthanasia and fixed in Bouin’s fixative at 4 °C for 24 h (Liu et al., 2016). Tissues were dehydrated through graded ethanol solutions, embedded in paraffin, sectioned (5 μm thickness), and mounted onto glass slides. Following deparaffinization and rehydration, sections were stained with hematoxylin-eosin (H&E) and periodic acid–Schiff (PAS) for histological evaluation.

### 2.10 Transmission electron microscopy (TEM)

Human nasal mucosal ciliated epithelial cells, and murine tissues and spermatozoa samples were fixed with 2.5% glutaraldehyde (Servicebio) followed by osmium tetroxide post-fixation. After dehydration through graded ethanol series, samples were embedded in resin, and ultrathin sections (∼80 nm thickness) were stained with lead citrate and uranyl acetate. Images were obtained using a Hitachi HT7700 transmission electron microscope equipped with a MegaView III camera (Olympus).

### 2.11 Scanning electron microscopy (SEM)

Tracheal segments were isolated, fixed overnight in 2.5% glutaraldehyde, washed in PBS, dehydrated through graded ethanol series, and air-dried. Samples were coated with gold before imaging with a Hitachi SU8010 scanning electron microscope.

### 2.12 Ciliary density and lengenth was quantitatively assessed using SEM and H&E

Images were acquired under consistent magnification and tilt angle from at least five randomly selected epithelial fields per sample. The proportion of ciliated surface area was determined by threshold-based segmentation of ciliary tips in ImageJ, and expressed as the percentage of the total epithelial surface area or as the number of ciliary bundles per 10–20 µm along the epithelial edge, as previously described (O’Brien et al., 2004).

For ciliary length, paraffin tracheal sections were stained with H&E and imaged at 40× under identical settings. In ImageJ, a blinded assessor measured the visible length of clearly delineated motile cilia along the epithelial border using the straight-line tool after spatial calibration. Per mouse, ≥5 nonoverlapping fields were analyzed; 20–30 cilia/field were measured when morphology permitted.

### 2.13 Real-time quantitative PCR (RT-qPCR)

Total RNA from tissues of wild-type and *Lrrc56*-KO mice was extracted using an RNA extraction and purification kit (K0731, Thermo Fisher Scientific). Reverse transcription of approximately 1 µg RNA was performed with EZscript reverse-transcription reagent (EZB-RT2GQ, EZBioscience). Target gene expression was quantified by RT-qPCR and normalized to *Gapdh*. Primer sequences are provided in Supplementary Table S1.

### 2.14 Tandem mass tag–mass spectrometry (TMT-MS)

For proteomic profiling, testes from five wild-type (WT) and five *Lrrc56* ^−/−^ mice were homogenized in 8 M urea buffer, followed by protein reduction with tris(2-carboxyethyl) phosphine hydrochloride (TCEP) and alkylation with iodoacetamide (IAA). After centrifugation at 21,000 × g to remove cellular debris, the supernatants underwent buffer exchange, and protein concentrations were quantified by measuring absorbance at 205 nm with a NanoDrop 2000c spectrophotometer (Thermo Fisher Scientific, United States). Protein samples were adjusted to 2.2 μg/μL, followed by overnight digestion at 37 °C using trypsin (enzyme-to-protein ratio of 1:50).

Subsequently, tryptic peptides were labeled with 10-plex TMT reagents at a reagent-to-protein ratio of 1:1 (w/w) for 30 min. Equal quantities of labeled peptides were pooled and fractionated via basic reversed-phase liquid chromatography using a 50-cm C18 column. Peptides were eluted over a 52-min gradient, and a total of 42 fractions were collected at 1-min intervals, which were subsequently concatenated into 10 fractions. Fractionated peptides were analyzed on an Orbitrap Fusion Lumos mass spectrometer as described previously (Zhou et al., 2023). Raw spectral data were processed using the IP2 pipeline, searched against the UniProt *Mus musculus* database (https://www.uniprot.org/proteomes/UP000000589), and filtered according to default parameters (Eng et al., 2015).

### 2.15 Statistical analyses

Statistical analyses were performed using SPSS version 17.0 and GraphPad Prism version 8.4.0. Descriptive statistics are presented as mean ± standard deviation (SD) or as counts (n) and proportions (%). The chi-square test was used for categorical data, and Student’s t-test was used for continuous variables. A p-value less than 0.05 was considered statistically significant.

## 3 Results

### 3.1 Clinical characterization of proband

**FIGURE 1:**
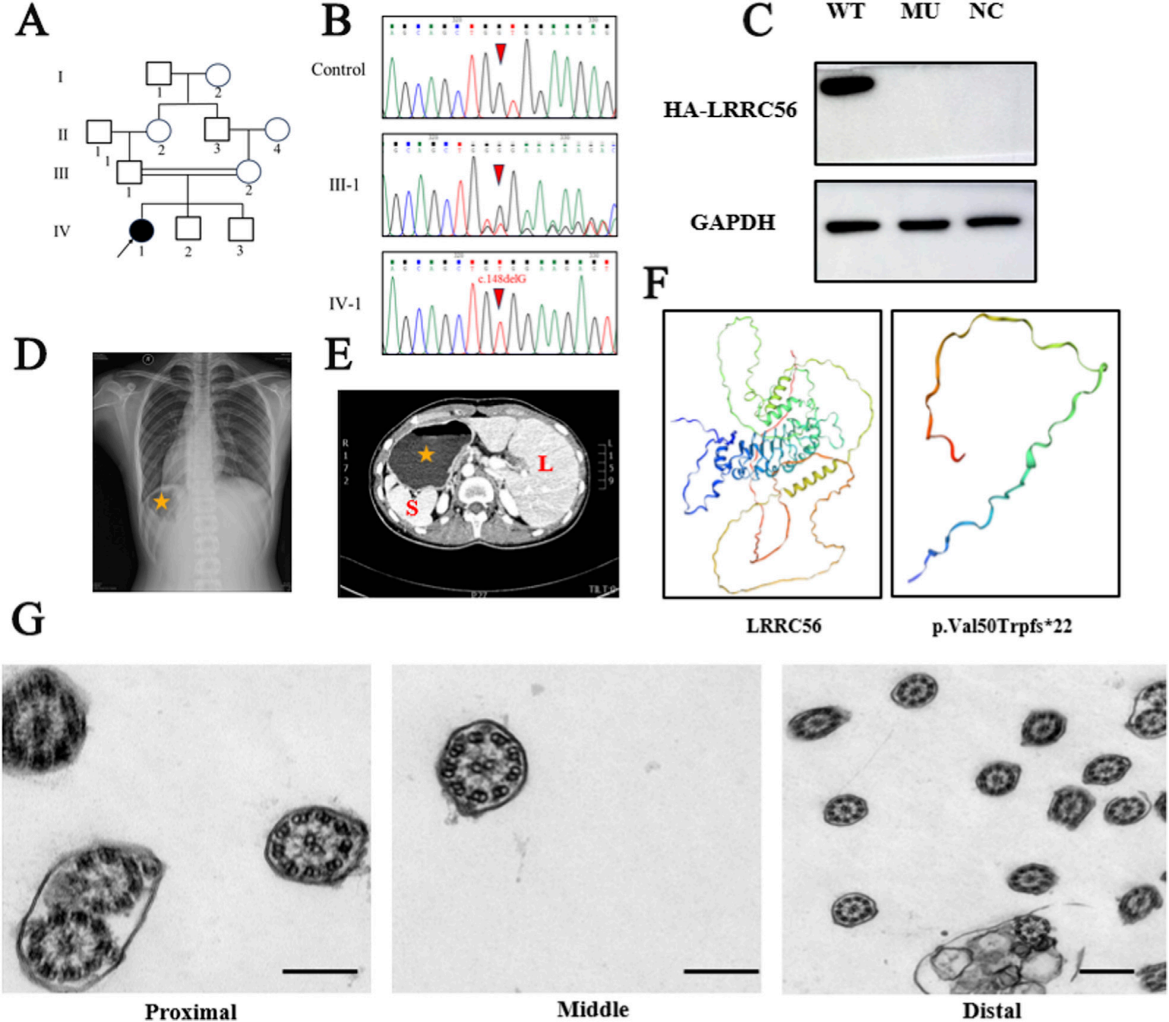
The clinical and genotypic features of the patient with LRRC56 homozygous variant. **(A)** Pedigrees of the family with inherited *LRRC56* pathogenic variant. Black arrow, proband. **(B)** Sanger sequencing chromatograms of the family with inherited *LRRC56* pathogenic variants (c.148delG) and normal control. **(C)** Chest X-ray of the proband showed dextrocardia, the gastric bubble is located on the right side of the body (indicated with yellow asterisk). **(D)** Abdominal CT of the proband showed abdominal situs inversus: liver in the left side (L: Liver), stomach and spleen in the right side (S: Spleen). **(E)** Overexpression of LRRC56-HA plasmids and LRRC56 mutant (c.148delG) plasmids, Western blot analysis of transfected 293T cells revealed a loss of LRRC56 mutant protein expression. WT: wild type, MU: mutation, NC: normal control. **(F)** SWISS-MODEL software predicted changes in the protein conformation of LRRC56 mutation. **(G)** Transmission electron microscopy (TEM) revealed intact 9 + 2 ciliary structure respiratory ciliated cells of the proband. bar:200 nm.

The proband originated from a family in which the parents were second-degree consanguineous relatives (Figure 1A). She was born at full term following an uneventful vaginal delivery. At 18 years of age, the patient presented to the hospital with acute abdominal pain and was subsequently diagnosed with cholelithiasis. During cholecystectomy, incidental findings of situs anomalies were noted, including situs ambiguus with mirror-image dextrocardia and a lobulated spleen (Figure 1C–D). Consequently, the patient was referred to our cardiovascular surgery department for further evaluation. Additionally, the patient reported mild chronic rhinitis with exacerbations characterized by severe coughing during upper respiratory tract infections, which necessitated intravenous antibiotic therapy. Her father had a medical history notable for chronic bronchiectasis.

Nasal mucosal ciliated cells were collected from the patient, and the general morphology and motility of motile cilia were analyzed by high-speed video microscopy. The mean ciliary beat frequency was 13.3 Hz, which was with normal limits (Supplementary Figure S1; Supplementary Table S3); Nevertheless, the ciliary beating pattern exhibited a rigid and restricted motion compared to normal control. (Supplementary Movies S1, S2). The patient’s nasal exhaled nitric oxide level averaged 300.5 nL/min, indicative of allergic rhinitis but not meeting the diagnostic threshold for PCD. The raw values, assay method, and reference ranges are provided in Supplementary Table S4. Transmission electron microscopy revealed that the 9 + 2 axonemal structure of the patient’s respiratory cilia was intact (Figure 1G).

### 3.2 Genetic analyses identified mutation in *LRRC56*

We subsequently recruited the patient into our ciliopathy cohort, which included a total of 121 individuals with motile ciliopathy. WES was performed to all 121 patient samples and further confirmed with a 284-gene transcriptome targeted sequencing panel associated with PCD (Paff et al., 2018). In the proband, we identified a frameshift mutation (c.148delG, p. Val50Trpfs22) in leucine-rich repeat-containing 56 (*LRRC56*; GenBank ID: NM_198075), which was confirmed by Sanger sequencing to segregate in an autosomal recessive manner within the family (Figure 1A IV-1; Figure 1B). The allele frequency of c.148delG (p. Val50Trpfs*22) is significantly less than 0.001 in population-based databases (e.g., 1000 G, ESP, ExAC, and gnomAD). This frameshift generates a premature termination codon at the 22nd codon within the shifted reading frame (p. Val50Trpfs*22), resulting in predicted truncation and likely loss of function of LRRC56. The mutation exhibited a high CADD score of 22.1, indicating its deleterious nature. Based on the American College of Medical Genomics (ACMG) standards and guidelines, this variant was classified as “likely pathogenic” (PVS1 + PM2), consistent with the patient’s observed phenotype.

To further evaluate the effect of the variant, we constructed wild-type *LRRC56* plasmids and *LRRC56* plasmids carrying the c.148delG mutation and transfected them into 293T cells. Both plasmids were tagged with an HA sequence. Western blot analysis showed a loss of protein expression in cells transfected with the variant (c.148delG) plasmid compared to those transfected with the wild-type plasmid. Overexpression in 293T cells suggested markedly undetectable steady-state levels of the mutant protein compared with wild type (Figure 1E). Given that 293T cells lack motile cilia, these data are interpreted as supportive of impaired protein translation rather than a definitive demonstration of loss of function. Using SWISS-MODEL software, structural modeling inferred that the Val50Trpfs22 variant resulted in a truncated LRRC56 protein (Figure 1F).

### 3.3 *Lrrc56*-deficiency in mice leads to laterality defects

**FIGURE 2:**
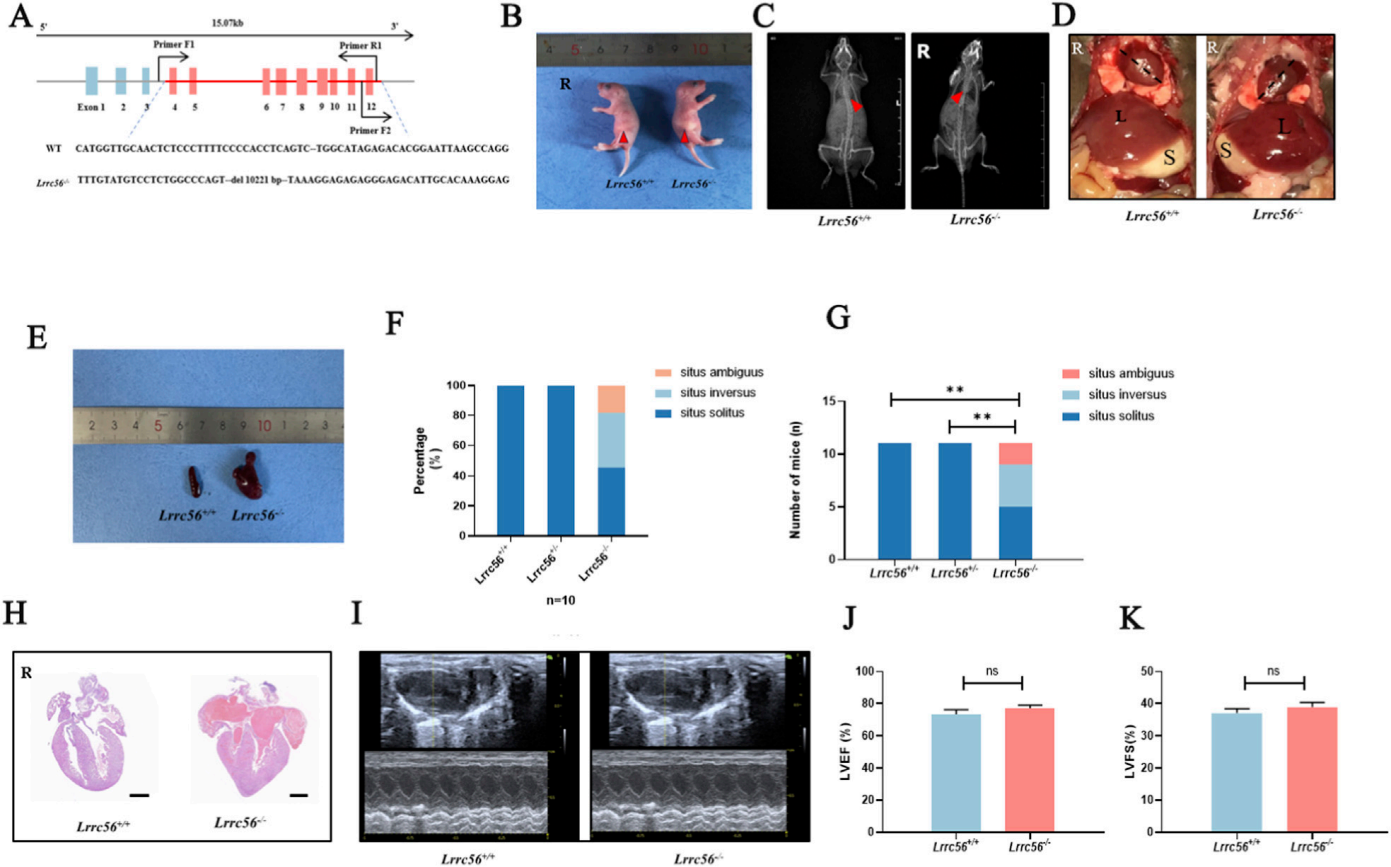
The knockout of *Lrrc56* leads to laterality defects in mice. **(A)** Schematic representation of the knockout strategy for generating *Lrrc^56−/−^* mice, involving the deletion of exons 4 -12. Genotyping primers Primer-F1, Primer-F2, and Primer-R1 were utilized. **(B)** *Lrrc^56−/−^* neonatal mice show the gastric bubble on the right side of the body, whereas in wild-type mice, the gastric bubble is located on the left side (indicated with the red arrow). **(C)** X-ray images of 8-week-old mice revealed in *Lrrc^56−/−^* mice, the apex of the heart is oriented towards the right side of the body, opposite to wild-type mice (indicated with the red arrow). **(D)** Anatomical diagrams indicate complete situs inversus in *Lrrc^56−/−^* mice. S: spleen, L: liver. The black dashed line signifies the cardiac axis direction. **(E)** Enlarged and lobulated spleen observed in *Lrrc^56−/−^* mice. **(F)** Statistical analysis of laterality defects in *Lrrc^56−/−^* mice shows approximately 50% presenting with lateral defects, including 30% exhibiting complete situs inversus (SI) and 20% showing visceral malposition (SA). **(G)** Numbers of mice with laterality defects were quantified in the *Lrrc^56+/+^*, *Lrrc^56+/−^*, and *Lrrc^56−/−^* groups. Pairwise comparisons (*Lrrc^56+/+^* vs. *Lrrc^56−/−^*; *Lrrc^56+/+^* vs. *Lrrc^56−/−^*) were conducted using χ^2^ tests; both comparisons showed significant differences (P < 0.01). **(H)** Histological staining of mouse heart sections (HE stain) reveals that in *Lrrc^56+/+^* mice, the apex of the heart is oriented towards the right side, with normal structures of major blood vessels and cardiac chambers, scale bar: 0.5mm. **(I)** Echocardiography demonstrates normal cardiac function in *Lrrc^56+/+^* mice. **(J)** Left ventricular ejection fraction (LVEF). The mean LVEF was 73% in wild-type mice and 77% in *Lrrc^56+/+^* mice. t-test, P>0.05, no statistically significant difference. **(K)** Left ventricular fractional shortening (LVFS) The mean LVFS was 37% in wild-type mice and 38% in *Lrrc* ^56*−/−*^mice. t-test, P>0.05, no statistically significant difference. P values: ** < 0.01, ** < 0.001, n.s indicates no statistically significant difference.

To investigate the functional role of LRRC56, we generated a loss-of-function Lrrc56 mouse model using CRISPR-Cas9 gene editing, as described in the Methods section. Exon 4 to exon 12 of the *Lrrc56* gene was targeted (Figure 2A), and the deletion was confirmed through sequencing and qRT-PCR analysis. Heterozygous *Lrcc56* mutant mice (*Lrrc56* ^*+/−*^) were viable fertile, and displayed no obvious phenotypic abnormalities. Homozygous *Lrcc56* mutant mice (*Lrrc56* ^*−/−*^) were generated by cross-breeding of heterozygous *Lrcc56* mutant mice.

*Lrrc56* ^*−/−*^ neonatal mice exhibited a right-sided gastric pouch, contrary to the normal left-sided arrangement (Figure 2B). X-ray imaging of 8-week-old wild-type and *Lrrc56* ^*−/−*^mice revealed rightward orientation of the cardiac apex in 50% of *Lrrc56* ^*−/−*^ mice, compared to the normal leftward orientation in all wild-type controls (Figure 2C). Further anatomical analyses confirmed the presence of laterality defects in *Lrrc56* ^*−/−*^ mice, including complete situs inversus and visceral heterotaxy (Figure 2D). Enlarged and lobulated spleens were also observed in some *Lrrc56* ^*−/−*^ mice (Figure 2E).

Out of the homozygous *Lrrc56* ^*−/−*^ mice examined (n = 11), approximately 54% (6/11) exhibited laterality defects. This included 36% (4/11) with complete situs inversus totalis and 18% (2/11) with visceral heterotaxy (Figure 2F). In contrast, none of the wild-type littermates (0/11) showed any laterality abnormalities. A chi-square test confirmed that laterality defects were significantly more frequent in *Lrrc56* ^*−/−*^ mice than in wild-type controls (p < 0.01) (Figure 2G). Despite the presence of dextrocardia, the cardiac structure, including major blood vessels and cardiac chambers, remained anatomically normal (Figure 2H). Echocardiography revealed no evidence of cardiac dysfunction in *Lrrc56* ^*−/−*^ mice (Figure 2I). Left ventricular ejection fraction (LVEF) and left ventricular fractional shortening (LVFS) were comparable to wild-type controls and remained within the normal range (Figures 2J,K).

### 3.4 *Lrrc56* deficiency leads to male infertility and MMAF

**FIGURE 3:**
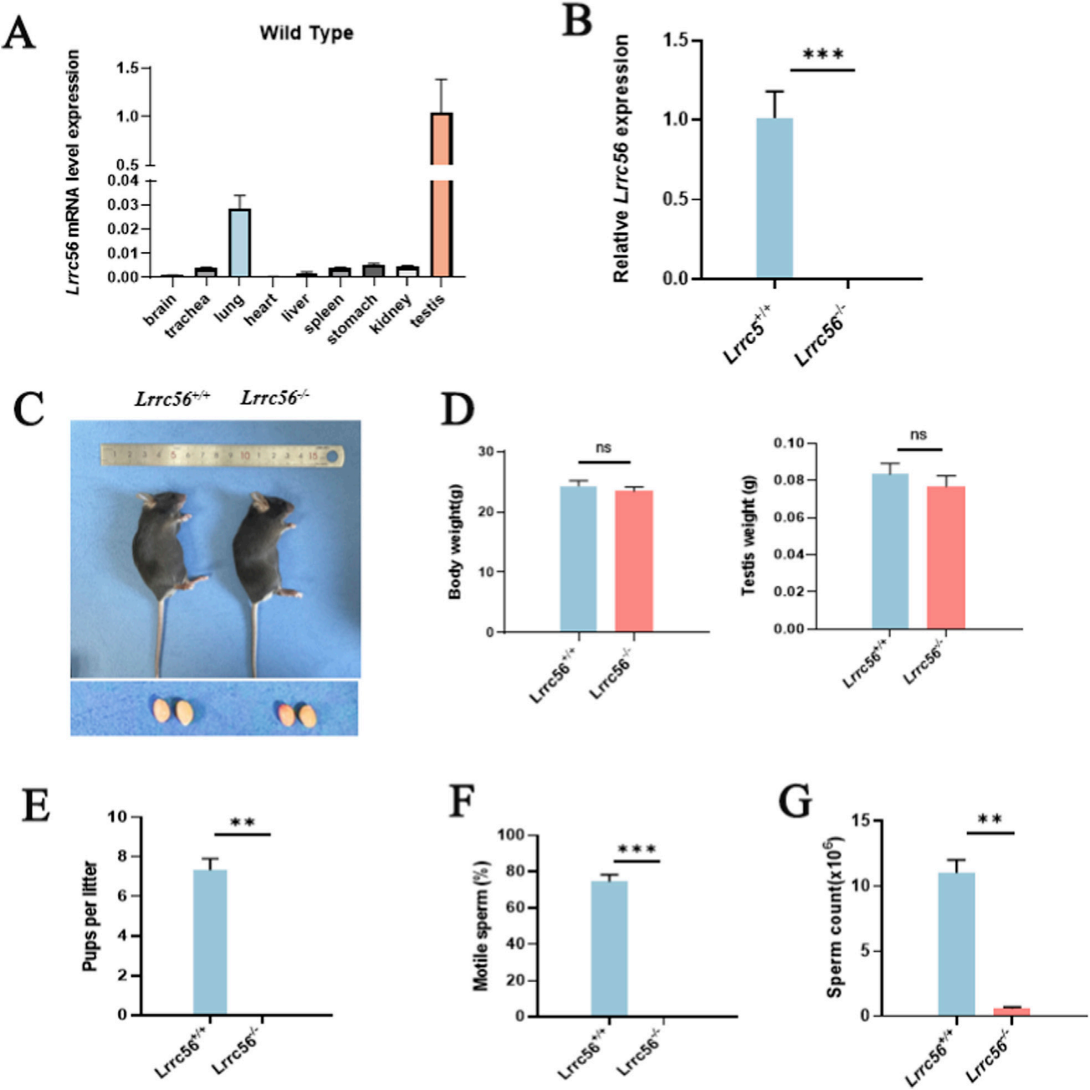
The knockout of *Lrrc56* leads to male infertility. **(A)** RT-qPCR results indicate predominant expression of Lrrc56 mRNA in mouse testes, lungs, and trachea. **(B)** Testicular *Lrrc56* mRNA levels. RT-qPCR shows that *Lrrc^56^* transcripts are nearly undetectable in the testes of *Lrrc* ^56*−/−*^ mice relative to wild-type controls. **(C)** External morphology and testicular appearance show no differences between *Lrrc* ^56*−/−*^male mice and *Lrrc* ^56*+/+*^ counterparts. **(D)** Body and testis weights. *Lrrc* ^56*−/−*^ mice exhibit no significant differences in body weight or testis weight compared with wild-type controls. **(E)** Fertility tests following mating with *Lrrc* ^56*+/+*^ female mice reveal that *Lrrc* ^56*−/−*^ male mice do not produce offspring. **(F)** *Lrrc* ^56*−/−*^ male mice exhibit complete absence of sperm. **(G)** Sperm from *Lrrc* ^56*−/−*^ male mice show complete immotility. t-test, P values: ** < 0.01, *** < 0.001, n.s indicates no statistically significant difference.

*LRRC56* expression was detected in multiple tissues, with the highest levels observed in the testis (Figure 3A). The successful generation of the *Lrrc56* knockout mouse model was confirmed at both DNA and mRNA level (Figure 3B). Gross examination of *Lrrc56* ^*−/−*^ adult (8 weeks) male mice revealed normal testis size and a normal ratio of testicular weight to body weight (Figures 3C,D). Although *Lrrc56* ^*−/−*^ males exhibited normal mating behaviors and produced coital plugs, they were infertile when paired with wild-type females (Figure 3E). Sperm samples from *Lrrc56* ^*−/−*^ males were subjected to Computer-Assisted Sperm Analysis (CASA), which revealed a significant reduction in sperm count compared to wild-type controls, along with complete sperm immotility in *Lrrc56* ^*−/−*^ samples (Figures 3F,G, Supplementary Movies S3, S4).

**FIGURE 4:**
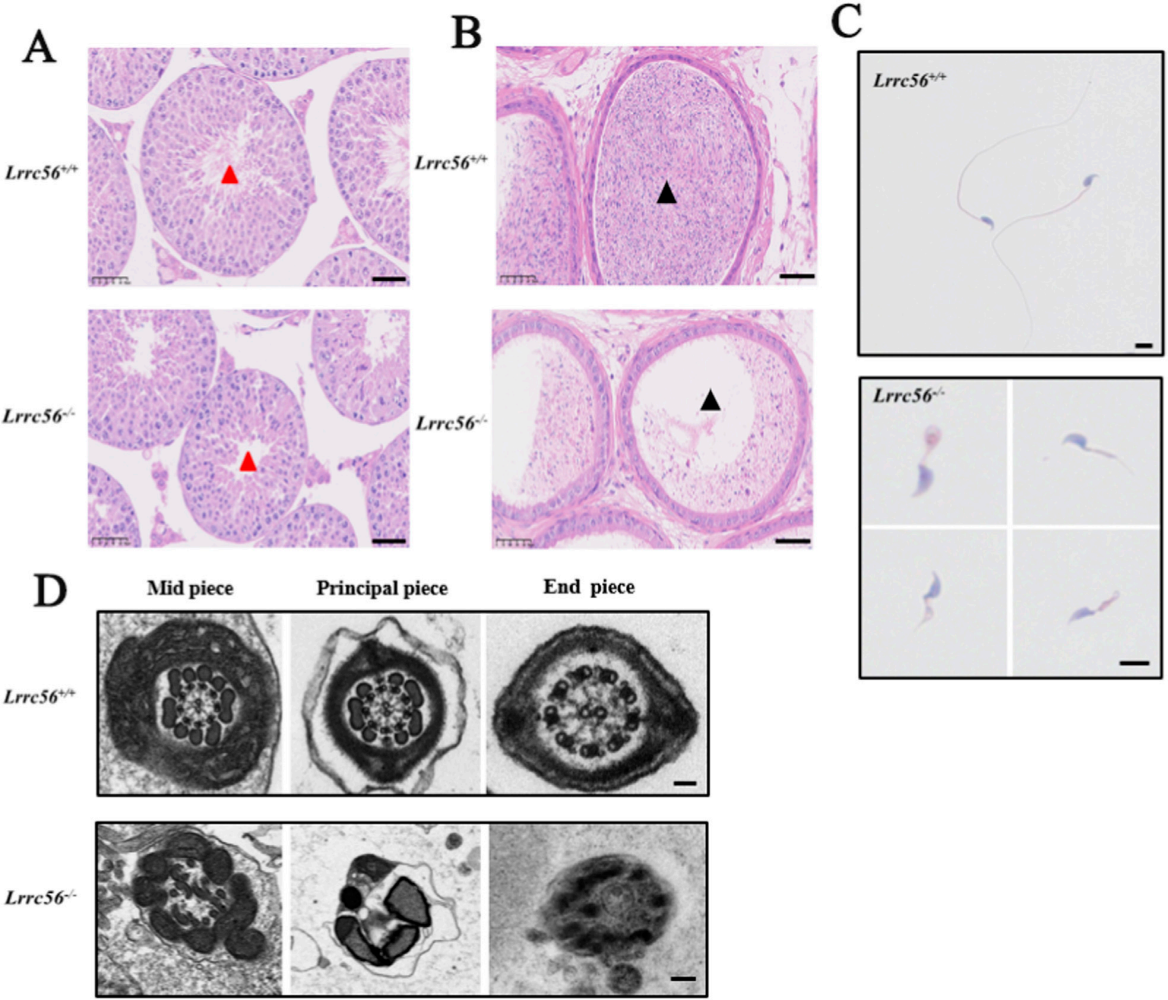
The knockout of Lrrc56 results in MMAF. **(A,B)** H&E staining of testis and epididymis in *Lrrc56* ^*+/+*^ and *Lrrc56* ^*−/−*^ mice. In *Lrrc56* ^*−/−*^ testes (red triangle markers) and epididymal lumens (black triangle markers), sperm flagella exhibit malformation and breakage, with fewer sperm having oval-shaped heads. Bar:50 μm. **(C)** Papanicolaou staining reveals various abnormalities in sperm flagella of *Lrrc56* ^*−/−*^ male mice, including tailless, short-tailed, coiled, and irregular tails; scale bar: 5 μm. **(D)** TEM images demonstrate the absence of a normal “9 + 2” structure in the axoneme of sperm flagella in *Lrrc56* ^*−/−*^ male mice; scale bar: 50 nm.

Histological analysis of the seminiferous tubules in *Lrrc56* ^*−/−*^ males showed aberrant flagellar structures compared to wild-type males, where tubular lumens typically contain flagella emanating from developing spermatids (Figure 4A). In addition, the epididymal lumen of *Lrrc56* ^*−/−*^ males was almost devoid of mature spermatozoa (Figure 4B). Morphological staining of sperm revealed significant abnormalities in flagellar morphology in *Lrrc56* ^*−/−*^ mice, including shortened tails, coiled tails, and irregular tail structures, compared to *Lrrc56* ^*+/+*^ mice (Figure 4C). Transmission electron microscopy (TEM) analysis showed that while *Lrrc56* ^*−/−*^ sperm retained a normal head structure, the 9 + 2 axonemal arrangement of the sperm tails was distinctly disorganized, in contrast to the well-defined structure observed in wild-type sperm (Figure 4D). These findings indicate that the loss of *Lrrc56* results in multiple morphological abnormalities of the flagella (MMAF) and an asthenospermia-like phenotype in male mice.

### 3.5 *Lrrc56* deficiency leads to hydrocephalus

**FIGURE 5:**
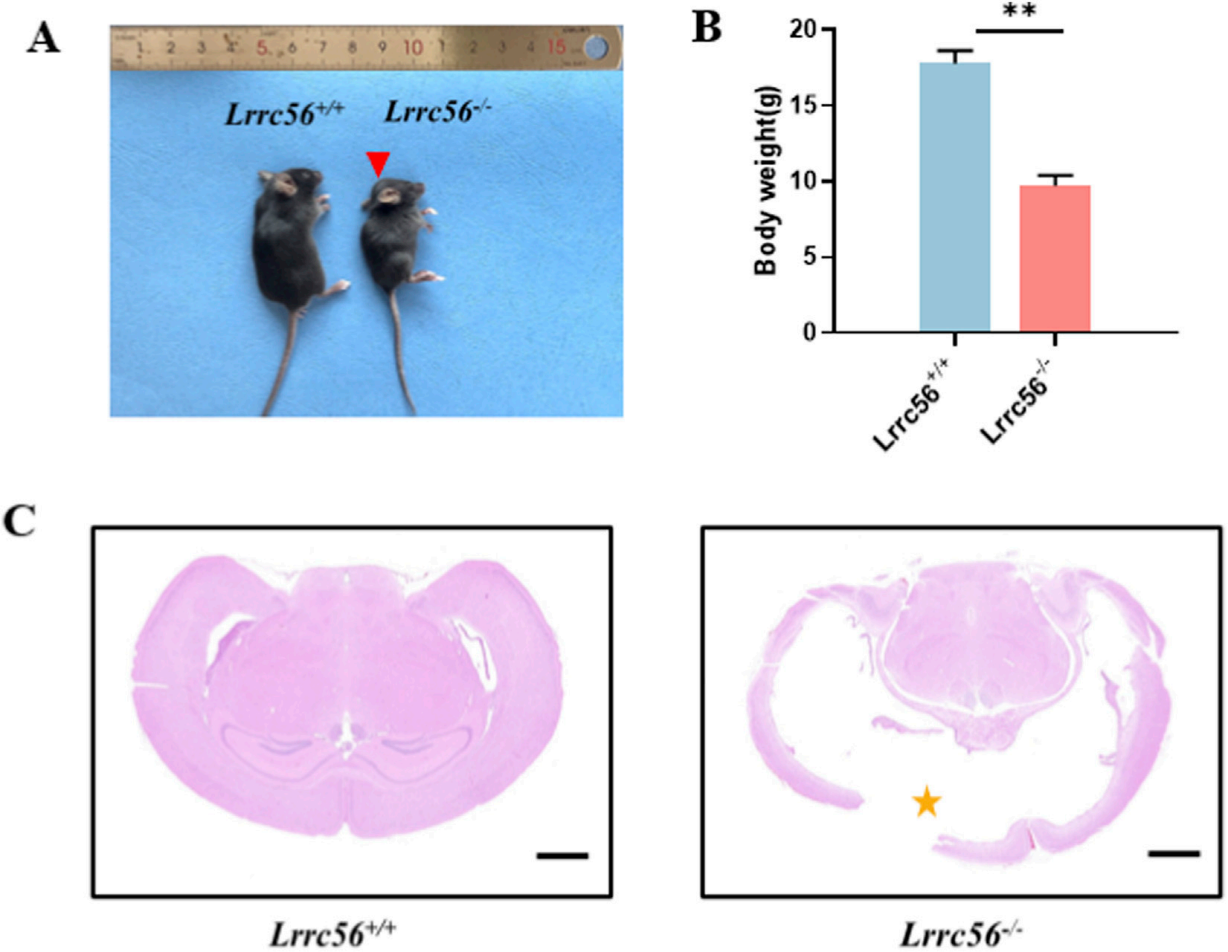
*Lrrc56* knockout leads to hydrocephalus. **(A)** Head photographs of *Lrrc56* ^*+/+*^ and *Lrrc56* ^*−/−*^ mice at 8 weeks of age. Compared with *Lrrc56* ^*+/+*^ male mouse, the head of *Lrrc56* ^*−/−*^ male mouse is larger and rounder with a bulging forehead (red arrow). **(B)** *Lrrc56* ^*−/−*^ mice exhibit noticeable weight reduction compared to *Lrrc56* ^*+/+*^ mice, with a prominent frontal protrusion. **(C)** Brain H&E staining of *Lrrc56* ^*+/+*^ and *Lrrc56* ^*−/−*^ male mice at 8 weeks of age. Compared with normal brain ventricles and cortex, the ventricles of *Lrrc56* ^*−/−*^mice were considerably widened, and the brain cortex shrunk due to ventricular widening and compression (yellow asterisk). Scale bars, 1 mm t-test, P values: ** <0.01, *** <0.001, n. s indicates no statistically significant difference.

*Lrrc56* ^*−/−*^ mice were born according to the expected Mendelian ratios, suggesting that the targeted disruption of the *Lrrc56* allele did not result in embryonic lethality. However, by postnatal day 10 (P10), approximately 30% of *Lrrc56* ^*−/−*^ mice exhibited noticeable growth retardation, characterized by significantly smaller body size compared to wild-type littermates (Figure 5A,B). Additionally, these mice displayed pronounced frontal protrusion and abnormal gait (Supplementary Movies S5). Histological analysis further confirmed the presence of hydrocephalus, as evidenced by marked dilation of the lateral ventricles and reduced cortical thickness, indicative of aberrant cerebrospinal fluid flow (Figure 5C).

### 3.6 *Lrrc56* deficiency leads to mucociliary cleaeance

To further elucidate the role of LRRC56 in the respiratory system, we examined its subcellular localization in tracheal tissue. Immunofluorescence and immunohistochemistry consistently demonstrated co-localization of LRRC56 with tubulin in the cilia of tracheal epithelial cells (Supplementary Figure S2A,B).

**FIGURE 6:**
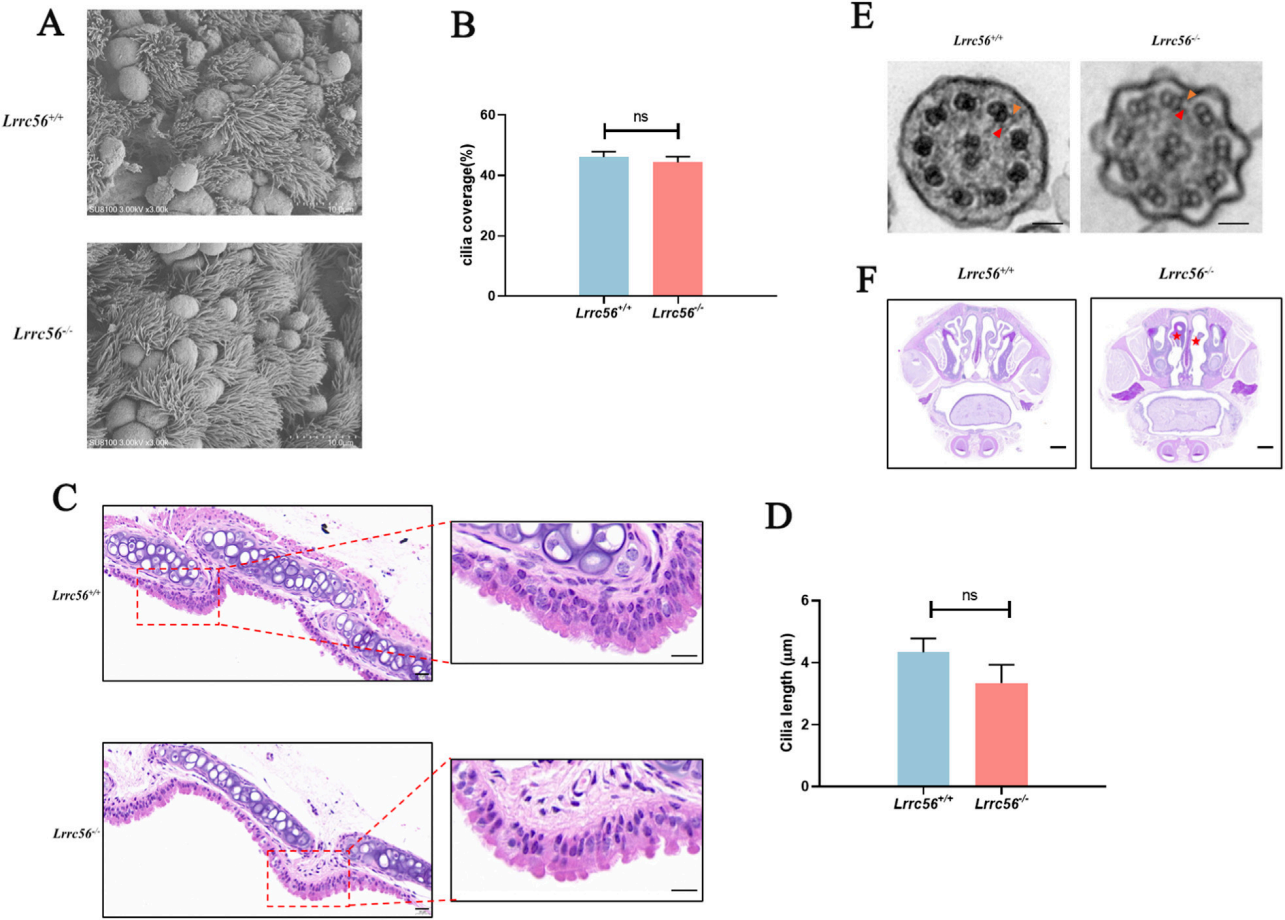
Effects of *Lrrc56* Knockout on cilia structure and function. **(A)** Scanning electron microscopy (SEM) of the tracheal epithelium at fixed magnification and tilt, showing the ciliated surface *Lrrc56* ^*−/−*^ mice compared to *Lrrc56* ^*+/+*^ mice. Bar:10 μm. **(B)** Quantification of ciliated-surface fraction (“coverage,” %) computed by ImageJ after background subtraction and adaptive thresholding of ciliary tip texture from ≥5 randomized epithelial fields per mouse. The mean ciliated-surface coverage of the tracheal epithelium was 46% in *Lrrc56* ^*+/+*^ mice and 44% in *Lrrc56* ^*−/−*^ mice. **(C)** H&E-stained tracheal epithelium from both mouse groups shows distinctly resolved cilia, suitable for ciliary length measurement. Bar:20 μm **(D)** Quantification of ciliary length (μm) derived from H&E staining of mouse tracheal sections used by ImageJ. The mean length of tracheal motile cilia (mean 3.3 ± 0.5 μm) in *Lrrc56* ^*−/−*^ mice was shorter than wild-type mice (4.3 ± 0.4 μm). (*Lrrc56* ^*+/+*^ n = 3, *Lrrc56* ^*−/−*^ n = 3. 5 fields/mouse, 20 total cilia measured). A t-test indicated significant difference (P = 0.01, <0.05). **(E)** Transmission electron microscopy of mouse tracheal cilia demonstrates the intact 9 + 2 structure in *Lrrc56* ^*−/−*^ mice. bar:50 nm. **(F)** PAS staining of mouse sinus sections showed that, compared with wild-type adult mice, *Lrrc56* ^*−/−*^ mice had significant mucus accumulation in the nasal cavity, indicating a loss of mucociliary clearance ability. Red asterisks indicate accumulated mucus. Scale bar, 1 mm t-test, P values: *<0.05, ** <0.01, *** <0.001, n. s indicates no statistically significant difference.

SEM revealed broadly comparable ciliary carpet coverage on the tracheal epithelium of *Lrrc56* ^*+/+*^ and *Lrrc56* ^*−/−*^ mice (Figure 6A). We quantified ciliated-surface fraction from at least five randomized fields per group: mean coverage was 46% in *Lrrc56* ^*+/+*^ and 44% in *Lrrc56* ^*−/−*^, with no significant between-group difference (Figure 6B). These data indicate that airway ciliary density is not reduced in the knockout. To assess ciliary length, tracheal sections were prepared and stained with hematoxylin and eosin (H&E), which facilitates measurement of individual cilia (Figure 6C). ImageJ-based quantification showed that mean tracheal ciliary length was 4.3 µm in *Lrrc56* ^*+/+*^ and 3.3 μm in *Lrrc56* ^*−/−*^mice. The statistical analysis (t-test) confirmed that cilia in *Lrrc56* ^*−/−*^ were significantly shorter than those in wild-type (Figure 6D).

Further examination through transmission electron microscopy confirmed that the cilia maintained the normal 9 + 2 microtubule structure in *Lrrc56* ^*−/−*^ mice (Figure 6E). Additionally, the nasal cavity of *Lrrc56* ^*−/−*^ mice manifested mucus accumulation, indicative of impaired mucociliary clearance (Figure 6F).

### 3.7 Proteomic analysis elucidating the function of LRRC56

**FIGURE 7:**
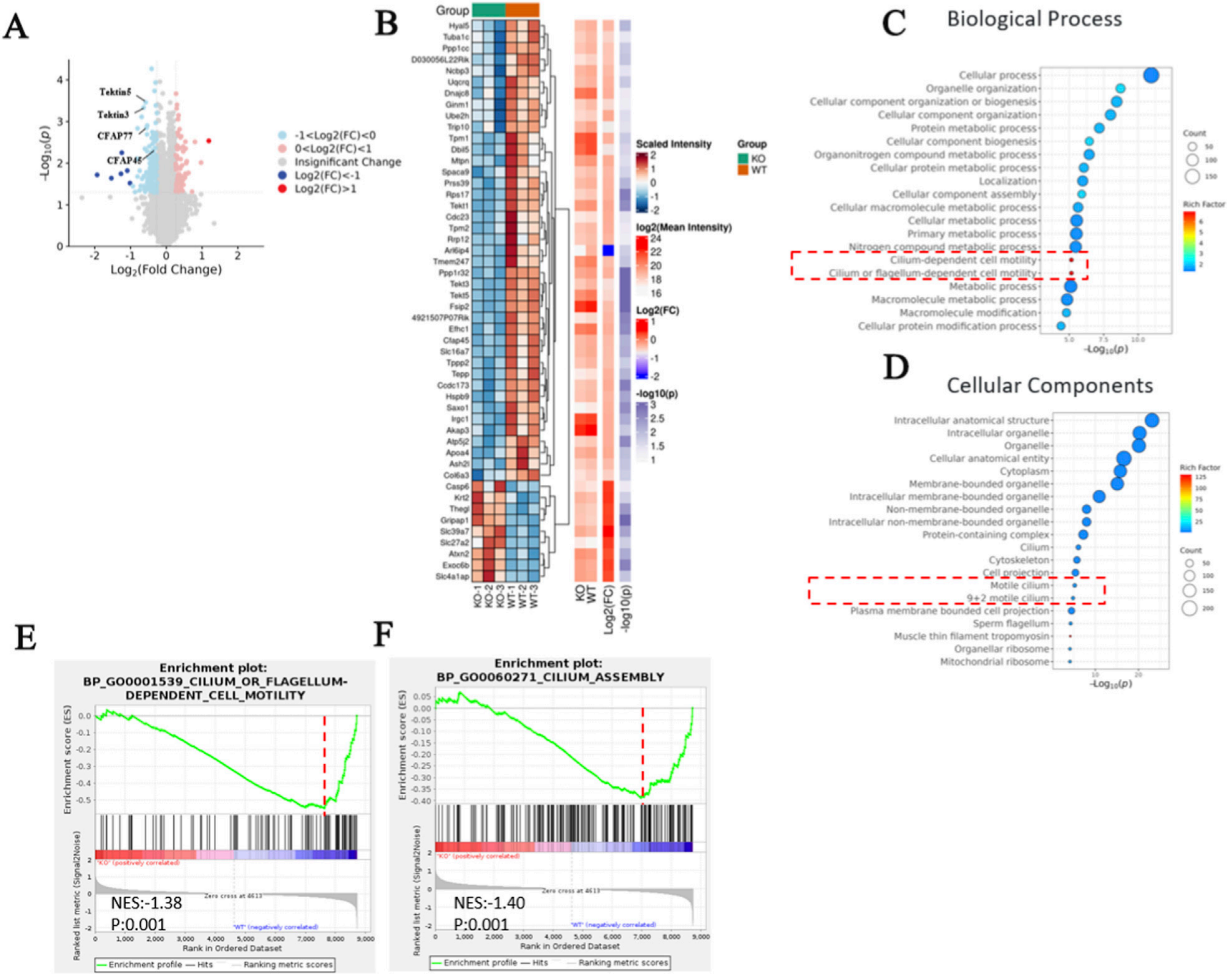
Proteomic Sequencing Analysis. **(A)** Volcano plot indicates upregulation of 76 proteins and downregulation of 154 proteins, with key downregulated proteins related to cilia annotated on the plot. Blue represents downregulated proteins, while red represents upregulated proteins. **(B)** Heatmap reveals the 50 proteins most correlated with cilia and flagella. **(C,D)** GO database analysis demonstrates enrichment of biological processes and cellular components related to cilia and ciliary movement. **(E,F)** Enrichment plots represent protein expressions of genes classified under these specific GESA terms; **(E)** motile cilia assembly and **(F)** cilium motility GESA terms were enriched in WT mice compared to those in *Lrrc56* ^*−/−*^mice.

To elucidate the role of LRRC56 in motile cilia function, we performed proteomic analysis using total protein extracts from testes of wild-type and *Lrrc56* ^*−/−*^ mice, employing 11-plex tandem mass tag mass spectrometry (TMT-MS). On average, more than 50,000 peptides and over 5,000 protein groups were identified per sample. After normalization, a total of 230 differentially expressed proteins (DEPs) were identified, with 76 proteins upregulated and 154 proteins downregulated (Figure 7A). Among the downregulated proteins, a significant subset was involved in the formation and function of microtubule inner proteins (Tektin5, CFAP77, CFAP45) and dynein arm assembly factors, such as LRRC6 (Figures 7A,B). To further illustrate the impact of LRRC56 deficiency on other ciliary structural proteins, we added a schematic of the motile cilium and annotated the key axonemal proteins identified by our proteomic analysis to their corresponding structural modules (Supplementary Figure S2).

Gene ontology analysis revealed that downregulated DEPs in *Lrrc56* ^*−/−*^ mice were associated with functions subsumed under the biological process (BP) category, particularly those related to cilium assembly and motility (Figure 7C). Furthermore, the DEPs were also linked to cellular components (CC), such as motile cilium and specific parts of cilium (Figure 7D). Gene set enrichment analysis (GSEA) further validated a general reduction in transcript expression of genes implicated in motile cilium assembly and cilium- or flagellum-dependent cell motility (Figures 7E,F).

## 4 Discussion

In this study, we characterized LRRC56 deficiency in humans and mice, providing evidence that mutations in *LRRC56* lead to a motile ciliopathy with clinical manifestations including situs anomalies (situs inversus totalis and heterotaxy), asthenospermia, hydrocephalus, and compromised mucociliary clearance. Notably, affected individuals exhibited mild respiratory symptoms but did not fulfill standard diagnostic criteria for PCD. Our findings are consistent with the initial report by Bonnefoy et al. describing *LRRC56* mutations in human PCD. Similar to their observation of marked ciliary motility defects with only subtle changes on conventional TEM, our *Lrrc56*-deficient model exhibits clear impairment of mucociliary clearance. Moreover, Bonnefoy et al. demonstrated that LRRC56 associates with IFT and is required for distal outer dynein arm assembly. This accords with our proteomic and phenotypic data and strengthens the view that LRRC56 functions as a dynein-arm assembly factor.

The proband carried a homozygous frameshift variant in LRRC56, predicted to disrupt protein function. Functional validation via *in vitro* assays suggests that this mutation is associated with a loss-of-function tendency. Clinically, the patient presented with laterality defects, recurrent upper respiratory tract infections, and rhinitis. HSVM revealed abnormal, rigid ciliary motility, with no major ultrastructural defects apparent on conventional TEM, indicative of primarily functional impairment. Previous reports described a homozygous c.494T>C mutation in *LRRC56* associated with situs inversus and severe congenital cardiac defects requiring extensive surgical intervention (Alasmari et al., 2022). However, our proband exhibited isolated mirror-image dextrocardia without cardiac structural anomalies, underscoring the phenotypic heterogeneity associated with *LRRC56* mutations.

Mice represent a critical model organism due to their genomic and physiological similarity to humans (Justice and Dhillon, 2016). In our study, *Lrrc56* ^*−/−*^ mice exhibited hallmark motile ciliopathy phenotypes, including situs abnormalities, asthenoteratozoospermia, hydrocephalus, and impaired mucociliary clearance. Interestingly, laterality defects were observed in approximately half of the *Lrrc56* ^*−/−*^ cohort, consistent with the stochastic nature of organ positioning previously documented (Nonaka et al., 2002; Hashimoto et al., 2010). Structural integrity of the cardiac chambers and great vessels was preserved in these mice, further supporting the hypothesis that LRRC56 primarily affects nodal ciliary function rather than directly influencing cardiac morphogenesis. Additionally, a large-scale ethylnitrosourea (ENU) mutagenesis screen did not implicate *Lrrc56* in CHD pathogenesis (Li et al., 2015).

We provide the evidence linking LRRC56 deficiency to male infertility characterized by immotile spermatozoa, reduced sperm counts, and multiple morphological abnormalities of the flagella. Unlike typical dynein arm defects, sperm flagella from *Lrrc56* ^*−/−*^ mice exhibited disrupted axonemal architecture, including loss of the canonical “9 + 2” configuration. Although murine respiratory cilia exhibited preserved axonemal integrity despite motility impairment, significant differences in dynein composition between sperm and respiratory cilia may explain these phenotypic discrepancies (Whitfield et al., 2019; Coutton et al., 2018).

Approximately 30% of *Lrrc56* ^*−/−*^ mice developed hydrocephalus, whereas no hydrocephalus was observed in the patient with biallelic *LRRC56* variants, highlighting species-specific differences and incomplete penetrance of motile-ciliopathy phenotypes. This divergence likely reflects neuroanatomical and physiological distinctions, such as the relatively shorter and wider human aqueduct of Sylvius, as well as compensatory cerebrospinal fluidcirculation or absorption, which together reduce susceptibility to obstruction despite ciliary dysfunction. (Ibañez-Tallon et al., 2003; Lee, 2011). These findings suggest that humans may possess compensatory mechanisms or anatomical adaptations that preserve CSF dynamics and prevent the onset of clinically overt hydrocephalus, even in the presence of biallelic *LRRC56* mutations. Moreover, hydrocephalus is frequently observed across murine motile-cilia gene knockouts (e.g., DNAH5, DNAAF1) but remains uncommon in human PCD except in rare ciliogenesis disorders (e.g., CCNO, FOXJ1), underscoring a rodent–human discrepancy (Sakamoto et al., 2021; Ha et al., 2016; Eisenhuth et al., 2025). Collectively, these findings indicate that LRRC56 deficiency results in a phenotypic spectrum, with hydrocephalus representing a manifestation predominantly observed in murine models (Wu et al., 2025). This species-specific expression underscores the need for caution when extrapolating ventricular phenotypes from mouse models to human patients, particularly in the context of motile ciliopathies.

In addition to hydrocephalus, growth retardation was observed in a subset of *Lrrc56* ^*−/−*^ pups during early postnatal development. Specifically, those presenting with hydrocephalus exhibited reduced body size and delayed weight gain during the neonatal period. This phenotype likely results from ventricular enlargement and elevated intracranial pressure, which may compromise feeding behavior or impose metabolic stress. However, among the mutants that survived beyond weaning, partial compensatory growth was evident. Notably, some mice with mild hydrocephalus attained near-normal body weights by 8 weeks of age, aligning with the weight distribution observed in adult mice. These findings indicate that early growth deficits in *Lrrc56*-deficient mice are primarily associated with severe hydrocephalus, while overall somatic development may normalize over time in less severely affected individuals.

We observed a shorter of tracheal motile cilia in *Lrrc56* ^*−/−*^ mice compared to wild type. A plausible mechanism is that LRRC56, the mammalian ortholog of ODA8, facilitates distal outer dynein-arm delivery via intraflagellar transport. Loss of LRRC56 would be expected to preferentially deplete distal ODA while leaving proximal cross-sections apparently intact on conventional TEM, which in turn destabilizes the distal axoneme and limits elongation (Bonnefoy et al., 2024; Bonnefoy et al., 2018; Balasubramaniam et al., 2024). This model aligns with human, algal, and protozoan data placing LRRC56/ODA8 on IFT trains and identifying it as essential for distal ODA assembly (Desai et al., 2015). Moreover, reduced IFT cargo loading or throughput is predicted to shift the balance between assembly and disassembly toward a shorter steady-state length, consistent with vertebrate studies linking IFT complex size and transport speed to ciliary length (Sun et al., 2014). In addition, maintenance of the distal segment, for example, septin-dependent tip stability, may be secondarily impaired, promoting tip erosion and further shortening (Kanamaru et al., 2022). Finally, because not all ODA or ODA-assembly defects shorten airway cilia across tissues or genotypes, these observations underscore tissue-specific expressivity and the need for direct tests such as distal ODA immunofluorescence mapping, live-cell IFT imaging, and electron tomography.

Proteomic analyses using liquid chromatography-mass spectrometry (LC-MS) on *Lrrc56* ^−/−^ mouse tissues revealed reduced expression of ciliary-associated proteins including microtubule inner proteins (MIPs) such as TEKT5, SPACA9, CFAP77, and dynein assembly factor LRRC6. These proteins are critical for maintaining structural connections within the axoneme (Gui et al., 2021; Walton et al., 2023). The observed preservation of the axonemal structure despite altered protein expression supports the hypothesis that LRRC56 functions predominantly in stabilizing protein-protein interactions essential for ciliary motility.

In conclusion, this study demonstrates that LRRC56 deficiency causes distinct motile ciliopathy phenotypes in humans and mice, revealing novel roles of LRRC56 in ciliary function and assembly. Our findings highlight the complex molecular mechanisms underlying ciliopathies and provide a foundation for further research aimed at exploring therapeutic interventions for ciliary disorders.

## 5 Limitations of the study

This study has several limitations. Firstly, because the patient declined repeated nasal brushings, we were unable to establish and Air–Liquid Interface (ALI)-differentiate nasal epithelial cultures. Consequently, post-culture assessments of ciliary motility could not be obtained. Moreover, we did not perform immunofluorescence co-localization of LRRC56 with dynein-arm markers in patient respiratory epithelial cells, which would directly demonstrate mislocalization or loss of key axonemal proteins in the absence of functional LRRC56. This was constrained by limited patient material. Second, the sample size is restricted to a single family (in addition to the mouse model), which may not capture the full phenotypic spectrum associated with LRRC56-related ciliopathy. Third, our proteomic analysis was conducted solely on testicular tissue and therefore primarily reflects effects on sperm flagellar proteins; these findings may not fully generalize to respiratory cilia or other motile ciliated tissues. Fourth, we have not yet performed rescue experiments by reintroducing LRRC56 to establish causality definitively. Nonetheless, the convergence of genetic, ultrastructural, and functional evidence supports a critical role for LRRC56 in motile cilia, and we explicitly acknowledge these limitations to guide future work.

## Data Availability

The datasets presented in this study can be found in online repositories. This data can be found in the ClinVar repository (https://www.ncbi.nlm.nih.gov/clinvar/) with the accession number SCV007097457, and the iProX repository (https://www.iprox.cn/) with the accession number PXD070799.

## Supplementary material

Supplementary Figure S1 LRRC56 subcellular localization in respiratory cilia. **(A)** Immunofluorescence of mouse respiratory cilia indicated colocalization of LRRC56 with acetylated TUBLIN on the cilia. bar: 50um **(B)** Immunohistochemistry results reveal the absence of LRRC56 expression on cilia in *Lrrc56* ^*−/−*^ mouse trachea, where LRRC56 typically colocalizes with acetylated TUBLIN. bar: 50um.

Supplementary Figure S2 Schematic representation of 9+2 ciliary structure components analysis.

Supplementary Table S1 Primers used in the study.

Supplementary Table S2 Antibodies used in the study.

Supplementary Table S3 Results of Respiratory Ciliary Beat Frequency (CBF) Measurement.

Supplementary Table S4 Results of Nasal Nitric Oxide (nNO) in the proband.
