# Supplementary figures and images for: LRRC56 deficiency cause motile ciliopathies in humans and mice
Source: Front Genet. 2025 Dec 17;16:1658063. doi: 10.3389/fgene.2025.1658063 (PMC12753093; doi:10.3389/fgene.2025.1658063)

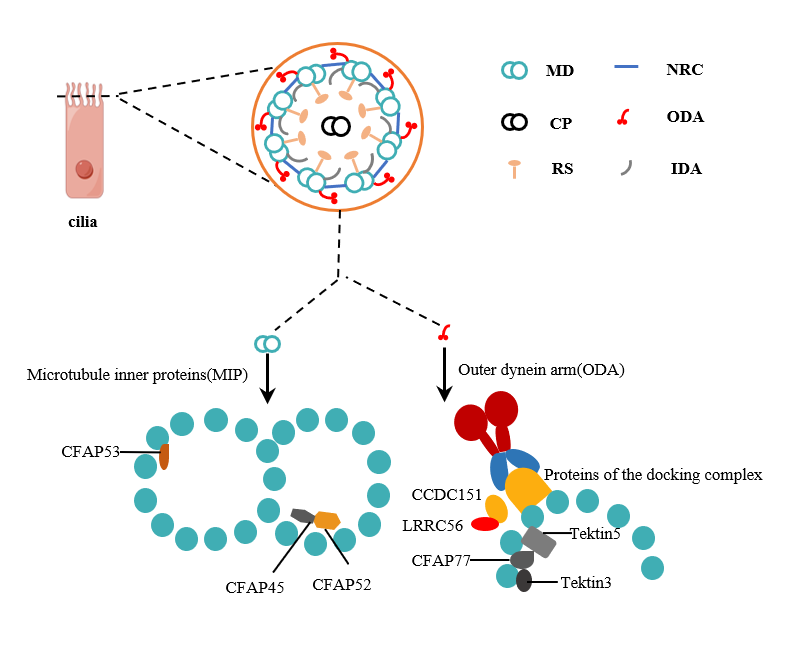

Supplement: Supplementary file 5 [file Image2.tif]

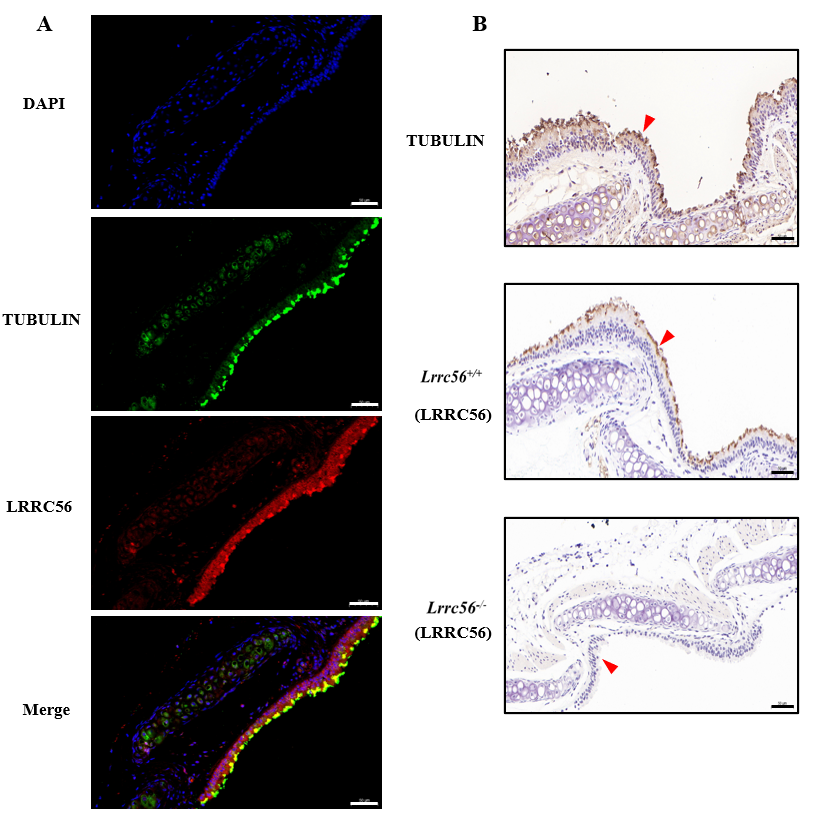

Supplement: Supplementary file 6 [file Image1.tif]
