# Supplementary material for: LRRC56 deficiency cause motile ciliopathies in humans and mice
Source: Front Genet. 2025 Dec 17;16:1658063. doi: 10.3389/fgene.2025.1658063 (PMC12753093; doi:10.3389/fgene.2025.1658063)
Supplement: Supplementary file 9 [file Table4.docx]

**Results of Nasal Nitric Oxide (nNO)**

| Parameter | Value | Unit |
| --- | --- | --- |
| Age / Sex | 18 years / Female |  |
| Method | Nasal exhaled NO (nNO), chemiluminescence |  |
| Ambient temperature | 27.1 | °C |
| Sampling flow | 0.6 | L/min (10 mL/s) |
| nNO concentration (mean ± SD) | 500.8 ± 11.7 | ppb |
| nNO production rate (mean ± SD) | 300.5 ± 7.0 | nL/min |
| Individual readings | 510.9, 487.6, 505.0, 514.8, 499.9, 486.9 | ppb |
| Interpretation* | Elevated nNO consistent with allergic rhinitis; does not meet PCD criteria |  |

*** Flow conversion: 10 mL/s equals 0.6 L/min; nL/min = concentration (ppb) × flow (L/min). Elevated nNO is compatible with allergic rhinitis and does not fulfill diagnostic criteria for PCD.
